# Selective Fiber Degeneration in the Peripheral Nerve of a Patient With Severe Complex Regional Pain Syndrome

**DOI:** 10.3389/fnins.2018.00207

**Published:** 2018-04-04

**Authors:** Adrien Yvon, Alessandro Faroni, Adam J. Reid, Vivien C. Lees

**Affiliations:** ^1^Nottingham University Hospitals, Nottingham, United Kingdom; ^2^Blond McIndoe Laboratories, Division of Cell Matrix Biology and Regenerative Medicine, Faculty of Biology Medicine and Health, Manchester Academic Health Science Centre, School of Biological Sciences, University of Manchester, Manchester, United Kingdom; ^3^Department of Plastic Surgery & Burns, Manchester Academic Health Science Centre, Wythenshawe Hospital, Manchester University NHS Foundation Trust, Manchester, United Kingdom

**Keywords:** complex regional pain syndrome (CRPS), peripheral nerve, transmission electron microscopy, nerve histology, remak bundle

## Abstract

**Aims:** Complex regional pain syndrome (CRPS) is characterized by chronic debilitating pain disproportional to the inciting event and accompanied by motor, sensory, and autonomic disturbances. The pathophysiology of CRPS remains elusive. An exceptional case of severe CRPS leading to forearm amputation provided the opportunity to examine nerve histopathological features of the peripheral nerves.

**Methods:** A 35-year-old female developed CRPS secondary to low voltage electrical injury. The CRPS was refractory to medical therapy and led to functional loss of the forelimb, repeated cutaneous wound infections leading to hospitalization. Specifically, the patient had exhausted a targeted conservative pain management programme prior to forearm amputation. Radial, median, and ulnar nerve specimens were obtained from the amputated limb and analyzed by light and transmission electron microscopy (TEM).

**Results:** All samples showed features of selective myelinated nerve fiber degeneration (47–58% of fibers) on electron microscopy. Degenerating myelinated fibers were significantly larger than healthy fibers (*p* < 0.05), and corresponded to the larger Aα fibers (motor/proprioception) whilst smaller Aδ (pain/temperature) fibers were spared. Groups of small unmyelinated C fibers (Remak bundles) also showed evidence of degeneration in all samples.

**Conclusions:** We are the first to show large fiber degeneration in CRPS using TEM. Degeneration of Aα fibers may lead to an imbalance in nerve signaling, inappropriately triggering the smaller healthy Aδ fibers, which transmit pain and temperature. These findings suggest peripheral nerve degeneration may play a key role in CRPS. Improved knowledge of pathogenesis will help develop more targeted treatments.

## Introduction

Complex regional pain syndrome (CRPS) is a collection of painful conditions that are characterized by a continuing regional pain, disproportionate to the usual course of any known trauma or other lesion (Harden et al., 2007). The pain is not in a specific nerve territory and typically has a distal predominance of abnormal sensory, motor, sudomotor, vasomotor, and trophic findings. It commonly occurs after trauma, however in up to 9% of cases, there is no traumatic trigger (Baron et al., 2002). The incidence in Europe is 26/100,000 person-years (de Mos et al., 2007), and ~4% of patients sustaining wrist fractures will go on to develop CRPS in the following 4 months (Moseley et al., 2014). The diagnosis of CRPS is largely one of exclusions and based on clinical assessment. The International Association for the Study of Pain first established a set of diagnostic criteria for CRPS, which were then revised in 2003 and termed the “Budapest” criteria (Harden, 2005).

The pathophysiology of CRPS remains elusive, and consequently treatment options are still inadequate. Several pathophysiological hypotheses have been suggested: Central nervous system (CNS) reorganization (Maihöfner et al., 2007; Lebel et al., 2008; Cohen et al., 2013), inflammation (Huygen et al., 2004; Parkitny et al., 2013), neurogenic inflammation from neuropeptides (Birklein et al., 2001), peripheral nerve pathology (van der Laan et al., 1998; Geertzen et al., 2015), capillary dysfunction (Schattschneider et al., 2006; Tan et al., 2012), and autoimmunity (Blaes et al., 2004; Kohr et al., 2009; Dirckx et al., 2015). Concerning peripheral nerves, a decrease in myelinated nerve fiber density has previously been demonstrated (van der Laan et al., 1998), and an animal model has suggested it affects primarily large fibers (Guilbaud et al., 1993). Another human study has also shown histological evidence of nerve fiber loss and regeneration in CRPS nerves, with specific large nerve fiber loss when compared to controls (Geertzen et al., 2015). Several hypotheses for peripheral nerve damage in CRPS have been proposed: local pressure from oedema (Guilbaud et al., 1993), microvascular ischemia from oxidative stress(Coderre and Bennett, 2008), retrograde degeneration (Geertzen et al., 2015), neurogenic inflammation(Weber et al., 2001), and decreased metallothionein (oxidative stress) (Oki et al., 2012).

Treatment strategies are based on addressing individual symptoms with known existing therapies to alleviate burden of disease (Perez et al., 2010). These strategies include treating pain following the World Health Organization analgesic ladder, neuropathic pain with anticonvulsants, topical dimethyl sulfoxide, ketamine, nerve blocks, physiotherapy, and psychological support (Perez et al., 2010; Birklein et al., 2015). For intractable CRPS where pain therapies have failed, amputation has been shown to have better outcomes than non-amputation (Midbari et al., 2016). Outcomes measured included several widely used pain and depression questionnaires and indexes. In a case series of therapy resistant CRPS, amputation was also associated with positive outcomes however 24% had recurrence (Krans-Schreuder et al., 2012). Another case series highlighted a 77% rate of phantom pain at 1 year post amputation (Bodde et al., 2014). A systematic review on the topic concluded that decision-making for amputation still remains a complex process due to insufficient evidence, and unpredictable outcomes, however, it should not be ignored as an option (Bodde et al., 2011, 2014).

We describe a rare case of CRPS leading to forearm amputation and the subsequent histopathological study of peripheral nerve tissue from the amputate. Our results demonstrate a selective large fiber degeneration which is a novel finding and in keeping with current knowledge on CRPS pathophysiology.

## Materials and methods

### Ethical approval

This study was carried out in accordance with the recommendations of the National Research Ethics Committee, UK with written informed consent from the subject, in accordance with the Declaration of Helsinki. The protocol was approved by the National Research Ethics Committee, UK (NRES 13/SC/0499).

### Patient

The patient in our study was a 35-year-old female who sustained a low voltage alternating current (110–380 V) electrical injury to her left hand. Left forearm pain, paraesthesia and bluish skin discoloration were features from initial presentation. In the 3 weeks following injury, she developed progressive patchy paraesthesia and dysaesthesia predominantly in the median nerve distribution of the affected hand. An ultrasound showed a normal appearance of the median nerve and of structures within the carpal tunnel and distal forearm. Acute carpal tunnel syndrome was suspected as being responsible for at least part of the presenting symptoms. With the possibility of secondary median nerve compression related to the injury, a carpal tunnel release procedure was undertaken at 3 weeks post injury. The median nerve appearance was macroscopically normal. Postoperatively, some paraesthesia in the territory of the median nerve improved but forearm pain persisted.

From 1 month post injury, the patient's disease continued to progress; all movements and sensory stimulants to the affected hand were grossly intolerable and a diagnosis of CRPS was made. The diagnosis was made based on the patient fulfilling the Budapest criteria, as shown in Table 1.

**Table 1:** Diagnosis of Complex Regional Pain Syndrome in the studied patient.

**Budapest criteria**	**Features present in patient**
The patient has continuing pain which is disproportionate to the inciting event	Pain was of early onset, progressive, unremitting despite all targeted interventions and disproportionate
The patient has at least one sign in two or more of the following categories: sensory, vasomotor, sudomotor, motor	Allodynia and hyperalgesia, decreased range of motion in wrist, fingers and thumb despite intensive hand therapy inputs, major trophic changes including skin changes and development of raw areas
The patient reports at least one symptom in three or more of the same categories: sensory, vasomotor, sudomotor, motor	Altered sensibility to light touch, joint movement, and experiencing magnified pain stimulus to pinprick. Reported differences in color of the two forelimbs, intractable stiffness despite full compliance with physiotherapy regimen
No other diagnosis can better explain the signs and symptoms	No alternative diagnosis was found (and she also came under care of the regional pain clinic who concurred with diagnosis of CRPS)

From 1 to 10 months post-injury, the patient received specialist therapy and pain management including pharmacological agents such as gabapentin, paracetamol, ibuprofen, tramadol, amitriptyline, ketamine, pamidronate infusions, lidocaine plasters, nabiximols, and capsaicin cream, which all failed to give relief. Supraclavicular catheter blocks, transcutaneous electrical nerve stimulation (TENS), and hand physiotherapy were also ineffective. At 9 months post injury, the patient was experiencing spontaneous skin breakdown with weeping, cellulitis and was hospitalized as a result of these infections. She was unable to clean her skin due to excessive pain. By the end of this period the patient had developed a functional loss of the limb. The patient requested an amputation which was ultimately supported by the multidisciplinary team consisting of plastic surgeons and pain anesthetists. The potential for control of pain was a secondary aim, as the impact of amputation on pain could not be fully predicted.

Ten months post-injury, the patient underwent a below elbow amputation at the level of the proximal third of the forearm, with a dorsally based flap of intact skin used to resurface the residuum. Nine months post-amputation the patient has not had any recurrence of CRPS at the amputation site, which is fully healed. She has had a dramatic response in terms of relief of CRPS symptoms. She has regained her quality of life and is wearing a cosmetic artificial arm prosthesis, with plans for her to progress to use of a myoelectric prosthesis.

### Samples

**Figure 1:**
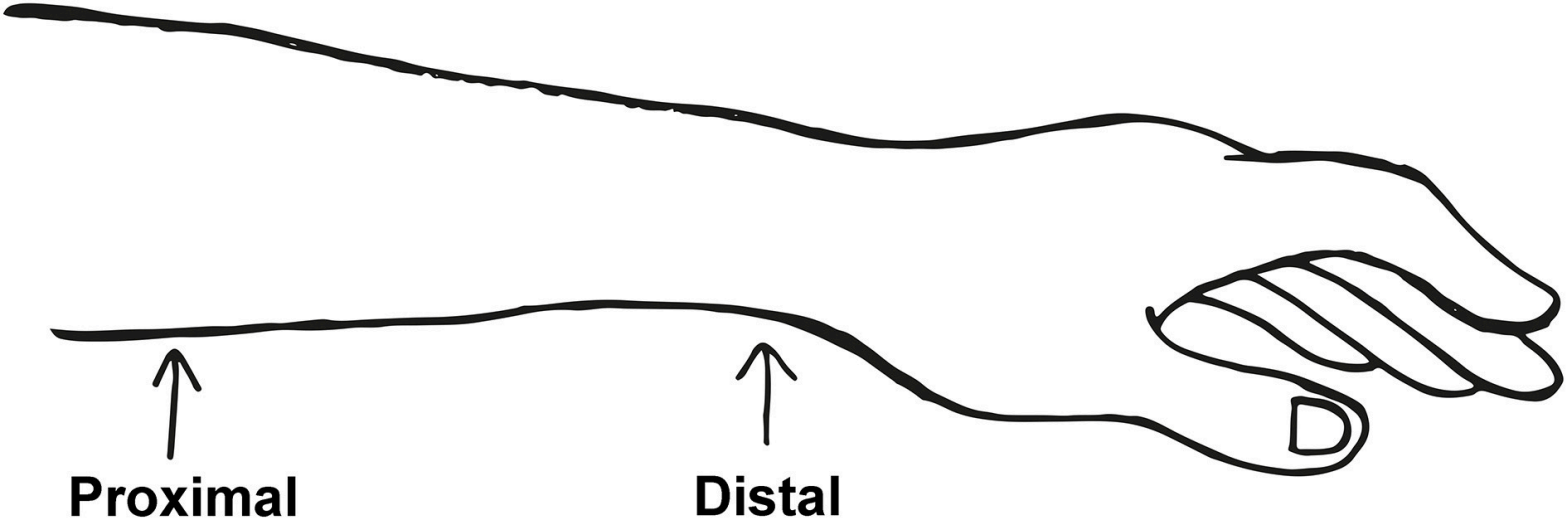
Diagram showing the level of forearm amputation as well as the levels at which the proximal and distal nerve samples were taken. Original biopsies were 3 cm long, which were further dissected during sample processing, fixation, and embedding for electron microscopy.

Nerve samples were obtained from the median, ulnar and radial nerves at the level of the distal and proximal thirds of forearm, as shown in Figure 1. Nerve biopsies of ~3 cm long were taken immediately after amputation through single transverse sharp excisions. The biopsies were further dissected into 1 cm long segments in order to allow fixation by immersion in 2% (w/v) paraformaldehyde and 2% (w/v) glutaraldehyde prepared in phosphate buffer saline solution. After primary fixation overnight at 4°C, nerve samples were further dissected in 3–4 mm long segments, reduced in Osmium (OsO4 1%) for 1 h, dehydrated and embedded in resin. Nerve samples were then cut into semi-thin sections (100–150 nm) for light microscopy and ultra-thin sections (50 nm) for electron microscopy.

### Electron microscopy

Transmission electron microscopy (TEM) images were obtained using a FEI Tecnai12 BioTwin microscope and images were taken using a Gatan Orius sc1000 digital Camera available at the Faculty of Biology, Medicine and Health Core Facilities, University of Manchester. For each nerve (distal and proximal samples combined), 18–20 images at x440 magnification were taken in different areas of the nerve fascicles chosen at random. Images were analyzed with ImageJ 64 imaging software (National Institutes of Health NIH, Bethesda, MD, USA). Nerve fibers were categorized according to status (healthy vs. degenerative) based on histological appearances. Histological features of fiber degeneration included distortion of myelin sheaths, entire fiber degeneration, degenerating Remak bundles, and denervated Schwann cell bands (unmyelinated axon loss). Nerve fiber size was measured according to maximum Feret diameter including myelin. Individual nerve fibers were measured and categorized according to the nerve fiber type classification, originally described by Gasser (1941), and depicted in Table 2 (adapted from Snell, 2010).

**Table 2:** Nerve fiber classification.

**Fiber type**	**Fiber size (μm)**	**Function**
Aα	12–20	Somatomotor, proprioception
Aβ	5–12	Touch, pressure
Aγ	3–6	Muscle spindle
Aδ	2–5	Pain and temperature
B	<3	Preganglionic autonomic
C	0.4–1.2 (unmyelinated)	Postganglionic autonomic, pain, temperature

*Adapted from Snell (2010)*.

### Light microscopy

After collection on glass slides, light microscopy semi-thin sections were further stained with toluidine blue 0.5% w/v for 60 s on a warm hot plate before mounting and analysis. Images were acquired with an Olympus IX51 inverted microscope (Olympus, Southend-on-Sea, UK). Light microscopy images were also analyzed with ImageJ 64 software. A total of 4 nerve fascicles for each nerve (2 distal and 2 proximal) were analyzed and myelinated nerve densities calculated and compared to findings from the literature.

### Statistical analysis

Statistical analysis comparing healthy and degenerative nerve fiber sizes was conducted using unpaired *t*-tests on Microsoft Excel 2007, as data was parametric. *P* < 0.05 were considered statistically significant.

## Results

Routine hospital histology of skin, muscle, fat, and forearm nerve specimens showed only mild superficial chronic inflammation in the superficial dermis and several foci of calcified material in the muscle.

### Myelinated nerve fiber counts

**Figure 2:**
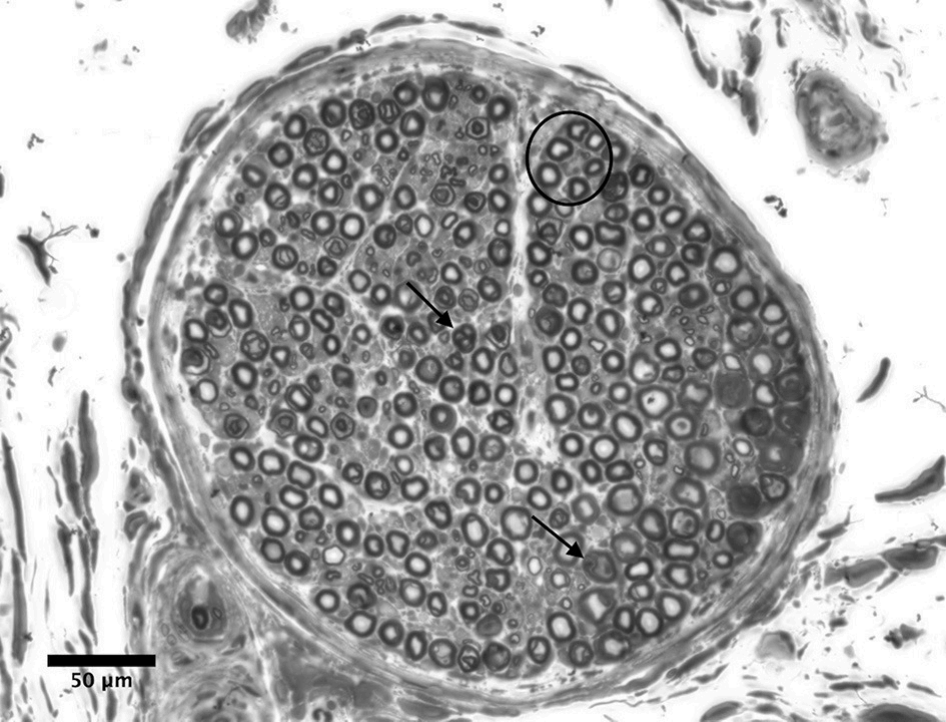
Light microscopy of a nerve fascicle from the distal ulnar nerve sample (bar represents 50 μm). The perineurium and endoneurium are visualized. The circle indicates a cluster of healthy myelinated axons. Arrows indicate examples of degenerating fibers. Degenerative features included myelin breakdown and collapsed nerve fibers. Stain: toluidine blue.

A total of *n* = 11,301 myelinated nerve fibers for radial, median and ulnar nerve samples were counted using light microscopy (Figure 2). Figure 2 depicts an entire nerve fascicle, with evidence of myelinated nerve fibers. Myelinated nerve fiber densities were calculated and compared to other CRPS affected nerves from the literature (Geertzen et al., 2015; Table 3). In addition, radial myelinated nerve fiber density was compared to healthy nerves from the literature (O'Sullivan and Swallow, 1968; Table 3); the medians of radial nerves in our study and healthy radial nerves in O'Sullivan and Swallow were similar. Furthermore, light microscopy also demonstrated the presence of nerve fiber degeneration in all nerve fascicles, although this was examined in greater detail using electron microscopy.

**Table 3:** Median myelinated fiber densities expressed (fibers/mm^2^).

**Median myelinated fiber densities expressed (fibers/mm^2^)**			
Nerve	This study (*n* = 1)	CRPS nerves (Geertzen et al., 2015)	Healthy nerves (O'Sullivan and Swallow, 1968)
Ulnar	6,208 (5,380–7,950)	5,400 (3,670–8,179)	–
Median	8,017 (5,652–9,656)	6,920 (5,662–8,284)	–
Radial	6,950 (6,443–8,490)	4,823 (4,194–7,025)	7,120 (5,410–10,020)

*Range expressed in brackets. Geertzen et al. include four CRPS patients (two male and two female) aged 23, 39, 44, and 45 years old. O'Sullivan and Swallow included eight patients with no peripheral neuropathy with a mean age of 40 years (range 17–57). The median of radial nerves in our study and healthy radial nerves in O'Sullivan and Swallow were similar*.

### Selective degeneration of myelinated nerve fibers

#### Myelinated nerve fibers

**Figure 3:**
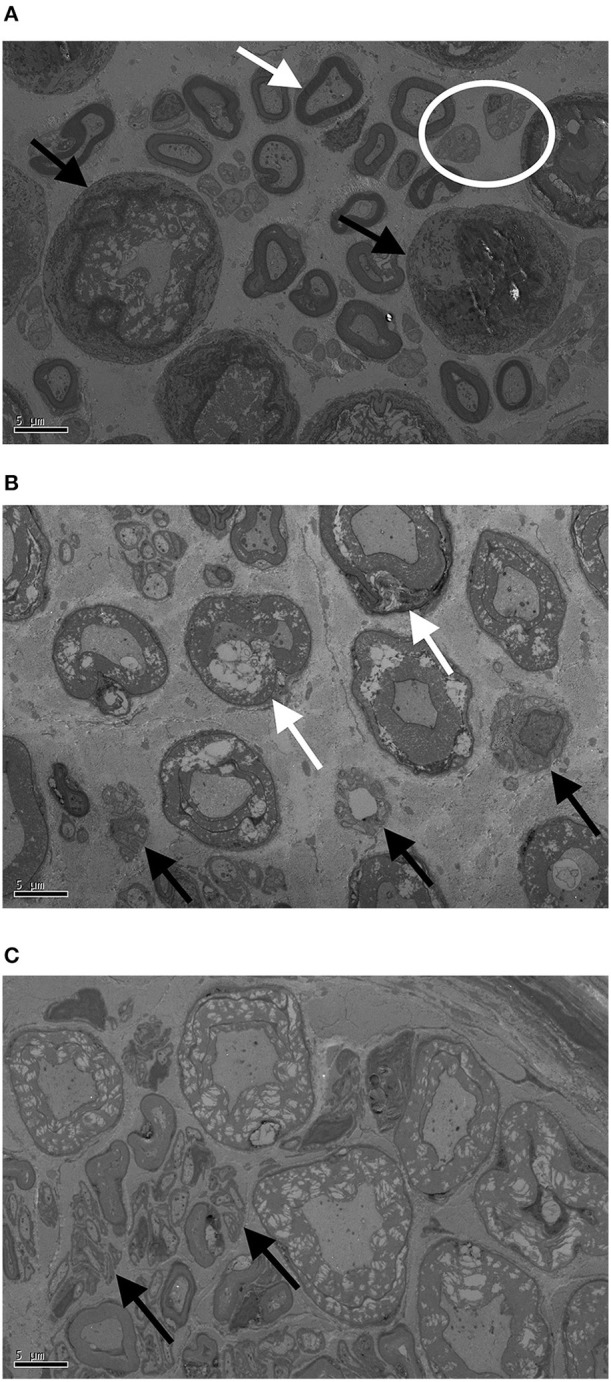
Transmission electron microscopy (TEM) images of proximal ulnar **(A)**, distal radial **(B)**, and proximal radial **(C)** nerve samples. Bar represents 5 μm. White arrow in **(A)** indicates healthy nerve fiber with Schwann cell, black arrows indicate entire fiber degeneration, and the white circle shows healthy Remak bundles (groups of unmyelinated fibers). In **(B)** white arrows show degenerating myelin while black arrows show degenerating Remak bundles. In **(C)** black arrow indicate denervated Schwann cell bands (unmyelinated axon loss).

**Figure 4:**
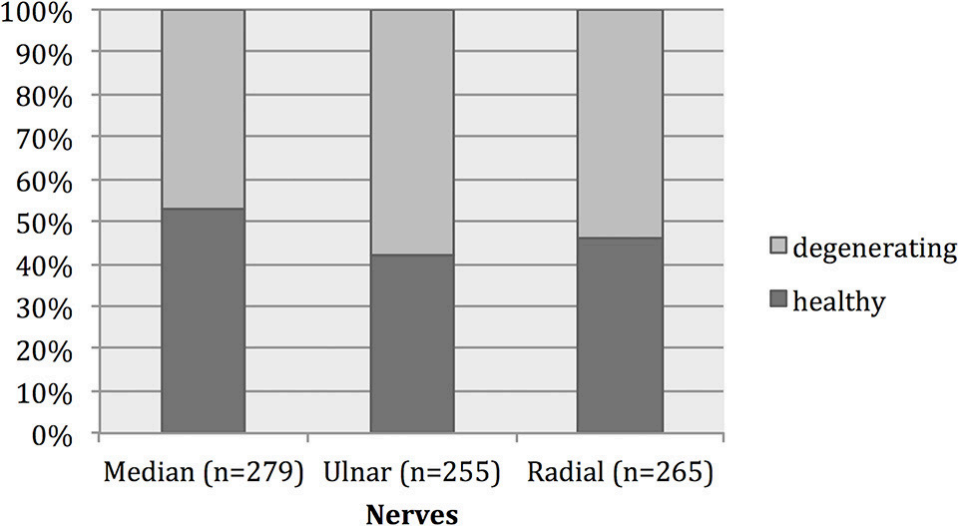
Histogram showing the percentage of healthy and degenerative myelinated nerve fibers in our study. Forty-seven to fifty-four percent of myelinated nerve fibers showed evidence of degeneration.

All nerve samples showed evidence of selective myelinated nerve fiber degeneration using electron microscopy (Figures 3A–C). No differences were found in fiber sizes or extent of degeneration between proximal and distal samples and these were thus analyzed together. Overall, 47–54% of myelinated nerve fibers showed evidence of degeneration (Figure 4).

**Figure 5:**
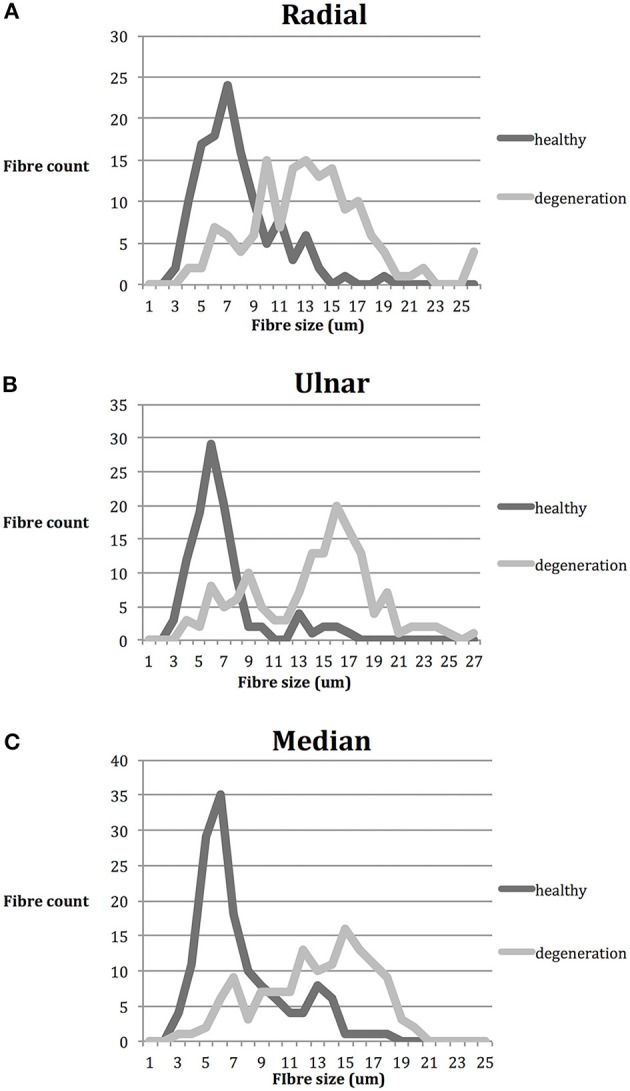
Nerve fiber distribution according to size (μm). *n* = 796. Gray: healthy; black: degenerative. **(A)** Radial nerve; smaller fibers were largely spared from degeneration, whilst the larger fibers are affected **(B)** Ulnar nerve; similar distribution to radial nerve, with two clear peaks of healthy and degenerative fibers **(C)** Median nerve; similar distribution to both radial and median nerve. The peak of healthy fibers count corresponds mainly to Aδ and Aβ fibers whilst the degeneration peak corresponds to Aα fibers.

A total of 796 nerve fibers for radial, ulnar, and median nerves were individually measured. Degenerating myelinated nerve fibers were significantly larger than healthy nerve fibers in all nerve samples (*p* < 0.01; Table 4). The healthy nerve fibers corresponded mainly to Aδ fibers and Aβ fibers whilst the degenerating fibers corresponded mainly to the Aα fibers. This was the case in all three nerves and their distribution is depicted in Figures 5A–C. In all samples Aδ were largely spared of degeneration.

**Table 4:** Mean myelinated fiber sizes in our study (μm).

	**Mean myelinated fiber size (μm)**		
	**Median (*n* = 279)**	**Ulnar (*n* = 255)**	**Radial (*n* = 265)**
Healthy	7.0 (SD 3.2)	6.3 (SD 3.0)	7.1 (SD 2.8)
Degenerating	12.3 (SD 3.9)	13.6 (SD 4.9)	12.6 (SD 4.4)
p-value	<0.05	<0.05	<0.05

*SD, Standard deviation*.

#### Unmyelinated nerve fibers

All nerve samples showed evidence of unmyelinated nerve fiber degeneration. Histological features of unmyelinated nerve fiber degeneration included degenerating Remak Bundles, and denervated Schwann cell bands (unmyelinated axon loss; Figures 5B,C). Between 16 and 43% of Remak Bundles showed evidence of degeneration on TEM (Table 5).

**Table 5:** Absolute counts of healthy and degenerating Remak bundles (groups of small unmyelinated C fibers) in all nerves in our study.

**Remak bundles (absolute counts)**		
	**Healthy**	**Degenerating**
Median	127	25
Ulnar	129	26
Radial	72	55

## Discussion

We describe ultrastructural changes in nerve fibers in the context of a rare case of CRPS requiring amputation of the distal forelimb. Results indicate that degeneration occurred selectively in large myelinated and small unmyelinated nerve fibers as compared to the medium and small myelinated nerve fibers, which were conserved. This has potential implications for the understanding of the pathogenesis of CRPS.

This study is believed to be the first showing ultrastructural images of large fiber (Aα) degeneration in CRPS using TEM. This was further corroborated by our light microscopy images showing large nerve fiber degeneration in all nerve fascicles. Our results are in line with a recent study of 15 severe CRPS patients who underwent amputation and where histological evidence of peripheral nerve pathology was found on light microscopy in radial, median, ulnar, tibial, and sural nerves (Geertzen et al., 2015). The Geertzen study found fiber loss in 81% of nerve biopsies and more importantly, it demonstrated a loss of large fibers (>12 μm) in sural nerves using light microscopy. We show the presence of histological degenerative features in large myelinated nerve fibers using TEM, thereby providing additional detail to that described by Geertzen et al. Similarly, an animal study of experimental pain, which used chronic loose ligatures of the sciatic nerve, also showed a selective loss of large fibers at 10 weeks, which did not affect small myelinated fibers (Guilbaud et al., 1993).

We propose that degeneration of Aα fibers may lead to an imbalance in nerve signaling if the majority of axonal messaging goes through the smaller healthy Aδ fibers, which may inappropriately trigger pain. As previously stated Aδ fibers are myelinated fibers that transmit pain and temperature and their hypersensitivity will cause symptoms of allodynia to light touch or temperature, or hyperalgesia. The concept of surviving fibers inappropriately firing when neighboring fibers are degenerating has long been established in neuropathic pain (Oaklander and Fields, 2009). In this respect, we propose treatment strategies for CRPS could potentially target the development of new drugs to modulate inappropriate firing of fibers (e.g., Pregabalin) and interventions that could decrease the extent of fiber degeneration.

Furthermore, we postulate that large Aα fiber degeneration contributes to motor symptoms in CRPS. As previously stated Aα fibers have a somatomotor function in the peripheral nervous system. Our patient suffered from motor weakness as CRPS progressed, and our histological findings are in keeping with this clinical picture. Movement disorders such as bradykinesia and dystonia affect around 25% of patients with CRPS (van Hilten, 2010). It could be that the range of large fiber degeneration in the disease predisposes patients to suffer from varying amounts of motor symptoms, depending on severity. Large fiber degeneration may consequently affect the associated innervated muscles. Evidence of atrophic muscular changes in CRPS has been shown in human studies (van der Laan et al., 1998; Hulsman et al., 2009). Muscular atrophy is also a well-known consequence of peripheral nerve degeneration in nerve injuries. Other hypotheses for motor dysfunction in CRPS which have been suggested include alterations in sensorimotor processing in the spinal cord (van Hilten, 2010) and disinhibition of the motor cortex (Schwenkreis et al., 2003).

Of interest, Geertzen et al. (2015) noted fiber regeneration in 86% of biopsies. In their study, they considered clusters of small myelinated fibers as evidence of regeneration. Whilst we also found these clusters in our electron microscopy analyses, we have not considered them as evidence of regeneration as these could also represent groups of smaller non-regenerating fibers. Histological evidence of regenerating fibers are generally viewed as fibers with thin myelin sheaths, as the regenerating fibers will initially lack myelin (Geuna et al., 2009). Whilst these were present in small frequencies in our TEM images, they were not a predominant feature. Furthermore, it is not certain that in CRPS simultaneous occurrence of both degeneration and regeneration takes place along the entire nerve.

Importantly this study has also demonstrated small unmyelinated C fiber pathology in upper limb CRPS nerves. This had previously been shown in 4 out of 8 patients in lower limb sural nerves (van der Laan et al., 1998). Partial C fiber degeneration can explain signs of autonomic dysfunction seen in CRPS, including swelling, skin, and temperature changes. Indeed, the concept of CRPS partly being a small fiber neuropathy has previously been described (Oaklander and Fields, 2009), and our results are consistent with this theory. This hypothesis is supported by studies examining skin biopsies of CRPS patients, which noted a decreased density of normal C fibers (Albrecht et al., 2006; Oaklander et al., 2006). In one of these studies (Oaklander et al., 2006), quantitative sensory testing (QST) was carried out in 344 CRPS patients, and small fiber afferent pathway dysfunction was seen in 39% of patients, whereas 48% had large fiber dysfunction. These QST results would be in keeping with our findings of simultaneous small fiber neuropathy and large fiber degeneration.

Radial nerve myelinated fiber densities in our study and healthy radial nerve densities in O'Sullivan et al. were similar, particularly when comparing the medians. Our results (Table 3) suggest myelinated nerve fiber densities were similar to both disease and disease-free controls. Although our study showed evidence of large myelinated fiber degeneration, this did not affect the fiber density. The reason for this is unclear. Similar findings have previously been reported, where evidence of nerve pathology did not translate to differences in nerve density (Geertzen et al., 2015). Myelinated nerve fiber density ranges considerably between individuals and is also known to decrease with age. In our study the patient's age was similar to the median ages of patients included in both studies used for comparison and therefore an overall comparison can be made. However, the low sample numbers (*n* = 1) means our myelinated nerve fiber density data should be interpreted with caution. The presence of potential regenerating fibers, as proposed by Geertzen et al. could also mean that nerve fiber loss is compensated for and densities consequently unaffected.

Limitations of this study are the sample size (*n* = 1) and the absence of a patient matched healthy nerve control, which are both dictated by the nature of the clinical case. Indeed, it would have been unethical and harmful to the patient to take a section of normal nerve from this patient's contralateral limb. Studying peripheral nerves in CRPS has long been challenging due to the limited scope of obtaining diseased nerve samples and healthy age-matched controls. For this reason there are very few studies in the literature that have examined peripheral nerves in CRPS (van der Laan et al., 1998; Oki et al., 2012; Geertzen et al., 2015). Furthermore, morphometric studies of healthy radial, ulnar and median nerves are also scarce, and it is difficult to draw comparisons with the limited data available in the literature. Amputated upper limbs are often secondary to vascular disease, extensive trauma, or oncological reasons where systemic and local factors may render these amputated limbs inappropriate to be truly considered as healthy controls. In addition, there is also no accurate or reliable animal model for CRPS.

Low voltage electrical injuries (<1,000 V) tend to not produce any neurological sequelae and rarely require clinical follow up or hospital admission. For this reason we know little about peripheral nerve changes following low voltage electrical nerve injury. It is unlikely our patient's condition was caused by low voltage electrical injury alone, without an additional pathological process such as that of CRPS. Electrical injury has been known to be a trigger for CRPS (Cohen, 1995). In a case series of 22 patients with low voltage electrical injury, 50% had immediate neurological symptoms, which resolved in 9 out of 11 patients (Grube et al., 1990). Another study of electrical injuries found a higher rate of numbness post injury in the low voltage group as compared to high voltage and said these symptoms appeared on average 2 months after the injury (Singerman et al., 2008). In one case series of mononeuropathies following burn injury (*n* = 32), low voltage electrical injuries were the cause of 50% of these whilst 43.7% of cases were caused by thermal burns alone (Tamam et al., 2013).

It is possible that our histological findings were due to chronic disuse of the upper limb alone, as our patient's nerves were examined at 10 months post injury. Immobilization and disuse are known factors contributing to CRPS, with one study reporting up to 47% of all CRPS sufferers as having a history of medically imposed immobilization (Allen et al., 1999). Another study found that hand surgery elective patients who wore a cast postoperatively had hypersensitivity and persisting pain for up to 1 month after cast removal (Pepper et al., 2013). This study also showed increased levels of inflammatory mediators in skin biopsies of these patients, similar to that seen in CRPS patients. However, there is relatively little literature on histological changes in disused peripheral nerves. In one animal study, disuse following tenectomy resulted in nerve fiber atrophy for “fast” muscles, whilst hemicordotomy resulted in an increase in diameter of nerve fibers supplying both “fast” and “slow” muscles (Calder and Pollock, 1985). In another study examining disused peripheral nerves following stroke, the frequency of abnormal teased nerve fibers was significantly increased with abnormal internodes frequently “clustered” and showing reduction in myelin thickness (Pollock et al., 1984). This same study also found the mean diameter of myelinated nerve fibers was reduced.

In summary, we demonstrate selective large myelinated fiber degeneration in the upper limb peripheral nerves of a patient with CRPS. We recognize the limitations of a single patient study and lack of control nerve examination; however, our findings have been placed into the context of all previously published literature and present an important hypothesis on the peripheral nerve pathophysiology of CRPS.

## Author contributions

All authors listed have made a substantial, direct and intellectual contribution to the work, and approved it for publication.

### Conflict of interest statement

The authors declare that the research was conducted in the absence of any commercial or financial relationships that could be construed as a potential conflict of interest. The handling Editor declared a shared affiliation, though no other collaboration, with several of the authors AY, AF, and AR.

## Abbreviations

CRPS: Complex regional pain syndrome
TENS: transcutaneous electrical nerve stimulation
TEM: transmission electron microscopy.
